# 7T clinical cardiovascular MR imaging: initial experience

**DOI:** 10.1186/1532-429X-14-S1-P234

**Published:** 2012-02-01

**Authors:** Hildo J Lamb, Linda Van Schinkel, Eleanore Kroner, Pieter J van den Boogaard, Maarten J Versluis, Albert de Roos, Andrew Webb, Hans-Marc J Siebelink

**Affiliations:** 1 Department of Radiology (C2S), Leiden University Medical Center, Leiden, Netherlands; 2 Cardiology, LUMC, Leiden, Netherlands

## Summary

Initial 7T Clinical Cardiovascular MR imaging results are presented. Although there are many basic problems that have to be resolved, 7T cardiovascular MRI has high potential for advanced imaging. Clinical practice has to prove what the specific advantages are of high-field cardiovascular MR imaging.

## Background

Recently, high-field 7T MR imaging was introduced. The higher field-strength has potential advantages, mainly related to increased signal-to-noise-ratio. One of the potentially exciting applications may be cardiovascular MR imaging. Cardiovascular MRI requires robust correction for heart motion, breathing motion and correction of field inhomogeneities. Therefore, cardiovascular imaging at high MR field strength has many challenges to be solved. Therefore, the purpose was to assess clinical feasibility of 7T cardiovascular MR imaging.

## Methods

Regular clinical patient exams are scheduled weekly at a regular time slot in our 7T MR ‘Gorter center’. During the initial phase of protocol development the presence of a dedicated MR physicist and MR technician is required, as well as presence of a cardiovascular radiologist and cardiologist. Clinical cardiovascular MRI was performed using a commercially available human whole-body 7T MRI system (Philips, The Netherlands), vector-ECG-gating and a custom-built 13-cm-diameter quadrature double-loop RF transmit-receive surface coil. Standard cardiovascular MR sequences were adapted from clinical 1.5T and 3T protocols. Breath-hold cine MR imaging was performed using TE 0.97 ms, TR 4 ms, flip angle 20, reconstructed pixel size 0.88x0.88x10 mm. Transmitral flow using velocity sensitivity of 150 cm/sec, TE 2.6 ms, TR 4.6 ms, flip angle 20, reconstructed pixel size 1.4x1.4x8 mm. Delayed enhancement acquisitions were performed approximately 15 minutes after intravenous administration of 0.1 mmol/kg Gadolinium (Dotarem, Guerbet) using an inversion-recovery 3D turbo-field echo sequence, TE 1.2 ms, TR 4 ms, flip angle 15, reconstructed pixel size 1.2x1.2x5 mm. Inversion time was determined with real-time plan scan to null normal myocardial signal. Vessel wall imaging was performed using TE 3.7 ms, TR 12 ms, flip angle 45, reconstructed pixel size 0.3x0.3x2 mm.

## Results

Initial clinical results are presented as a pictorial essay. Examples were acquired in a routine clinical setting. ECG-triggering was effective in about 90% of patients. See Figure for first clinical 7T results.

## Conclusions

7T clinical cardiovascular MR imaging is feasible. Many basic problems still have to be resolved, such as improved ECG-triggering, improved penetration depth of the transmit-receive surface coil, multi-transmit setup, optimalization of inversion pulses for delayed enhancement and vessel wall imaging. Clinical practice has to prove what the advantages are of high-field cardiovascular MR imaging.

## Funding

No disclosures.

**Figure 1:**
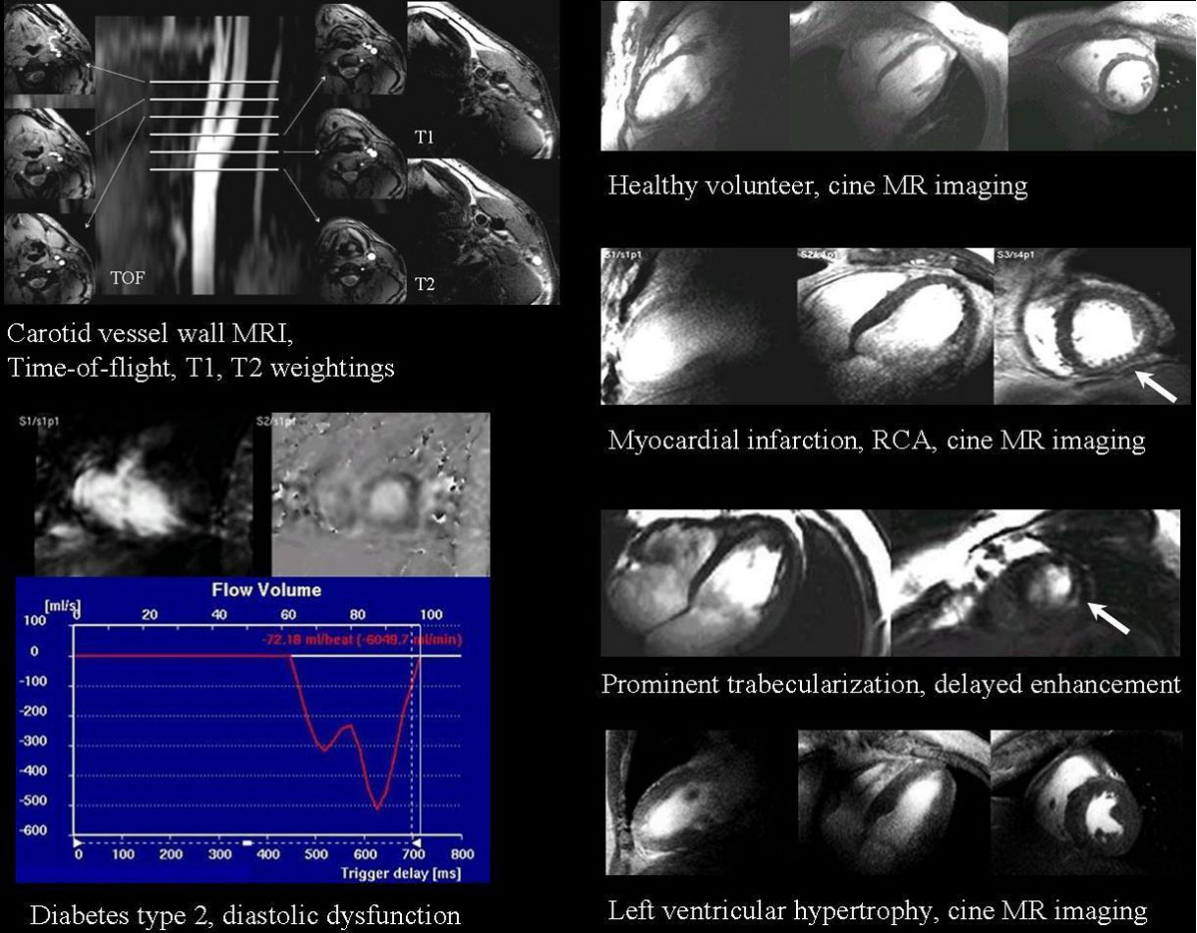
Examples of initial clinical experience with 7T Cardiovascular MR imaging. See text below images for clinical explanation, see abstract text for technical details.

